# A dog model for centronuclear myopathy carrying the most common *DNM2* mutation

**DOI:** 10.1242/dmm.049219

**Published:** 2022-04-14

**Authors:** Johann Böhm, Inès Barthélémy, Charlène Landwerlin, Nicolas Blanchard-Gutton, Frédéric Relaix, Stéphane Blot, Jocelyn Laporte, Laurent Tiret

**Affiliations:** 1 Médecine translationnelle et neurogénétique, Institut de Génétique et de Biologie Moléculaire et Cellulaire (IGBMC), Inserm U 1258, CNRS UMR 7104, Université de Strasbourg, 67404 Illkirch, France; 2 Neuroscience et psychiatrie, Université Paris-Est Créteil, INSERM, IMRB, 94010 Créteil, France; 3 Ecole nationale vétérinaire d'Alfort, IMRB, 94700 Maisons-Alfort, France; 4 EFS, IMRB, 94017 Créteil Cedex, France

**Keywords:** Neuromuscular disorder, Congenital myopathy, Dynamin, Large animal model, T-tubules, MTM1

## Abstract

Mutations in *DNM2* cause autosomal dominant centronuclear myopathy (ADCNM), a rare disease characterized by skeletal muscle weakness and structural anomalies of the myofibres, including nuclear centralization and mitochondrial mispositioning. Following the clinical report of a Border Collie male with exercise intolerance and histopathological hallmarks of CNM on the muscle biopsy, we identified the c.1393C>T (R465W) mutation in *DNM2*, corresponding to the most common ADCNM mutation in humans. In order to establish a large animal model for longitudinal and preclinical studies on the muscle disorder, we collected sperm samples from the Border Collie male and generated a dog cohort for subsequent clinical, genetic and histological investigations. Four of the five offspring carried the *DNM2* mutation and showed muscle atrophy and a mildly impaired gait. Morphological examinations of transverse muscle sections revealed CNM-typical fibres with centralized nuclei and remodelling of the mitochondrial network. Overall, the DNM2-CNM dog represents a faithful animal model for the human disorder, allows the investigation of ADCNM disease progression, and constitutes a valuable complementary tool to validate innovative therapies established in mice.

## Abstract (precis)

**Summary:** Description of a spontaneous dog model and generation of a cohort carrying the most common *DNM2* mutation associated with autosomal dominant centronuclear myopathy in humans.

## INTRODUCTION

Cellular membranes undergo constant shape remodelling through curvature, tubulation, constriction and fission to enable fundamental biological processes, such as cytokinesis, migration, endocytosis, phagocytosis, signalling, intracellular trafficking, recycling or compartmentalization (McMahon and Gallop, 2005). These dynamic events rely on the concerted interplay of lipids, proteins and the cytoskeleton, and one of the key factors of membrane remodelling is dynamin 2 (DNM2) (Ferguson and De Camilli, 2012). This ubiquitously expressed mechanochemical enzyme is able to reorganize microtubule and actin networks, and self-assembles into helical structures at the neck of nascent vesicles to induce vesicle release under GTP hydrolysis (Antonny et al., 2016; Chappie et al., 2009; Gu et al., 2010; Warnock et al., 1997).

Dynamin 2 is composed of an N-terminal GTPase domain, a middle domain (MID), a phospholipid-binding pleckstrin homology domain (PH), a GTPase effector domain (GED) and a C-terminal proline-arginine-rich domain (PRD) implicated in protein-protein interactions (Faelber et al., 2011; Ferguson and De Camilli, 2012). Mutations in the *DNM2* gene are associated with three distinct autosomal dominant neuromuscular disorders: Charcot–Marie–Tooth neuropathy (CMT1B, MIM# 606482), spastic paraplegia (Sambuughin et al., 2015) and centronuclear myopathy (ADCNM, MIM# 160150), the predominant centronuclear myopathy (CNM) form in adult patients (Bitoun et al., 2005; Zuchner et al., 2005). ADCNM is characterized by generalized muscle weakness, ptosis and ophthalmoplegia, and muscle biopsies from affected individuals typically show fibre size heterogeneity, abnormal nuclear centralization, mitochondrial mispositioning, and radial arrangement of sarcoplasmic strands (Bitoun et al., 2005). The age of onset and disease severity ranges from severe neonatal hypotonia and respiratory distress to mild adult-onset muscle weakness, and correlates with the position of the mutation. To date, more than 100 families and about 20 different ADCNM-related *DNM2* mutations have been reported, essentially clustering in hotspot regions in exons 8, 11, 14, 15 and 16 (Abath Neto et al., 2015; Bohm et al., 2012; Werlauff et al., 2015). The most common mutation c.1393C>T resides in exon 11, affects approximately 25% of the cases, leads to the amino acid substitution p.ArgR465Trp (R465W) in the MID domain, and most often results in a moderate clinical presentation involving childhood onset and slowly progressive distal muscle weakness (Bohm et al., 2012). In accordance with the clinical and histological presentation of the patients, the *Dnm2^R465W/+^* knock-in mouse model manifests reduced force associated with abnormal muscle structure and function (Durieux et al., 2010). Here, we describe a spontaneous canine model harbouring the DNM2 R465W mutation and the generation of a dog cohort with clinical and histopathological characteristics paralleling the human disorder.

## RESULTS

### Identification of the *DNM2* c.1393C>T (R465W) mutation in a Border Collie

**Fig. 1.**
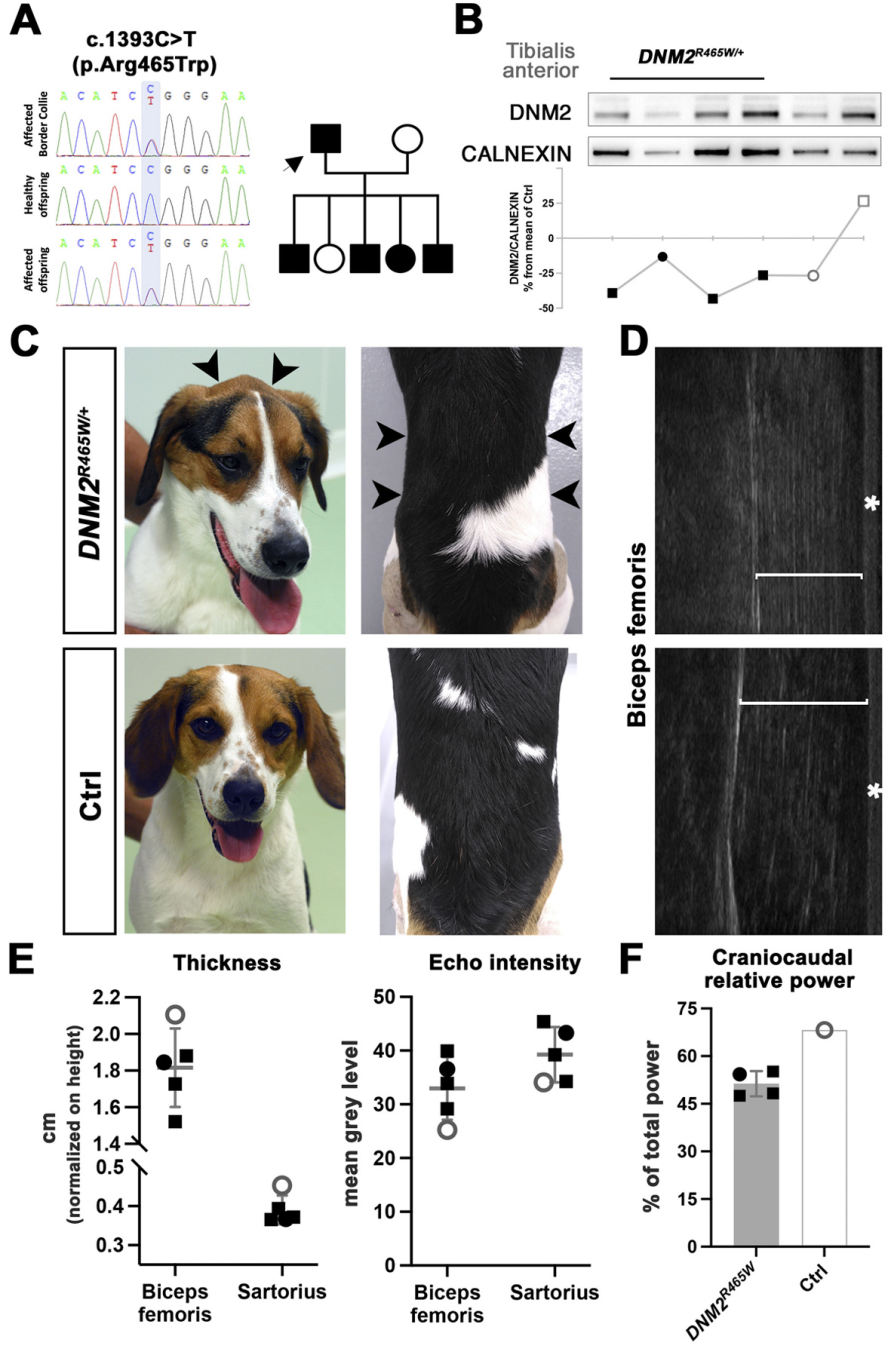
**Molecular, morphological and functional features of canine DNM2-CNM.** (A) Pedigree, segregation and electropherogram showing the *DNM2* mutation. Arrow indicates the Border Collie proband. (B) Western blot of tibialis anterior muscle extracts revealed DNM2 protein in affected dogs (lanes 1-4, black symbols), in the healthy littermate (lane 5, white circle) and in an age-matched Golden Retriever control (lane 6, white square). Calnexin served as a loading control. Quantification of the DNM2 protein level, normalized to calnexin, is shown below each lane. (C) Representative pictures of an affected dog and healthy littermate at 12 months of age. Note the marked atrophy of the masticatory and paraspinal muscles (arrowheads). (D) Ultrasound imaging providing a longitudinal view of the biceps femoris muscle and revealing reduced muscle thickness and an increased echo intensity in an affected dog. Asterisks mark the skin. (E) Graphs illustrating the reduced thickness and higher echo intensity values of the biceps femoris and sartorius muscles of the affected dogs. (F) Gait analysis by accelerometry at 12 months uncovered a lower relative craniocaudal power in all four affected dogs compared with the healthy littermate. Black squares represent affected males, black circle the affected female, white circle the healthy female littermate. The horizontal lines in E represent mean values of the four affected dogs. Error bars represent s.d.

A 2-year-old Border Collie male presented with a 1-year history of exercise-induced pelvic limb collapse and a short-strided and stiff gait, and morphological analysis of a muscle biopsy uncovered histopathological anomalies suggestive of CNM (Eminaga et al., 2012). We therefore Sanger sequenced the known canine CNM genes *HACD1* (previously named *PTPLA*), *MTM1* and *BIN1*. An insertion of the SINE retrotransposon in *HACD1* exon 2 is associated with CNM in Labrador Retrievers (Pele et al., 2005), and missense and splice site mutations in *MTM1* and *BIN1* were respectively shown to cause CNM in Labrador Retrievers, Rottweilers, Boykin Spaniels, and Great Danes (Beggs et al., 2010; Bohm et al., 2013; Olby et al., 2020; Shelton et al., 2015). However, no pathogenic DNA variant was found in these genes. Finally, Sanger sequencing of the autosomal gene *DNM2* on chromosome 20 disclosed the heterozygous c.1393C>T (R465W) transition in exon 11, corresponding to the most common ADCNM mutation in humans (Fig. 1A).

### Generation of a dog cohort: clinical and histopathological characterization

To confirm the pathogenicity of the identified *DNM2* mutation in dogs and to establish a large and relevant animal model for long-term studies on disease development and the evaluation of innovative therapeutic approaches, sperm was collected from the Border Collie male to inseminate a Beagle female. The resulting litter of two female and three male pups were genotyped and underwent thorough clinical follow-up over 12 months. Biopsies from the tibialis anterior and biceps femoris muscles, both easily accessible and extensively studied in CNMs, were taken at one year of age and used for protein extraction and histological investigations.

From the five offspring, four were found to carry the c.1393C>T (R465W) missense mutation in *DNM2* (Fig. 1A). In accordance with human patients harbouring the same R465W substitution (Bitoun et al., 2005), a western blot on muscle extracts detected the dynamin 2 protein in the dogs. Despite variable signal intensities among the samples, quantification revealed comparable dynamin 2 protein levels in affected and control dogs (Fig. 1B), confirming that the identified missense mutation does not impair mRNA or protein stability.

At 12 months of age, all *DNM2^R465W/+^* dogs showed general muscle atrophy particularly affecting the masticatory and paraspinal muscles (Fig. 1C). Transcutaneous ultrasound confirmed atrophy of the biceps femoris and sartorius cranialis muscles, and revealed enhanced echo intensities, suggesting an alteration of the muscle texture (Fig. 1D,E). The affected dogs had increasing difficulties with jumping and standing on the pelvic limbs (Movie 1), and gait analysis evidenced subtle anomalies and, notably, a reduced craniocaudal power pointing to a decreased forward propulsion (Fig. 1F). Complete blood counts and routine biochemistry profiles were within the reference ranges, and serum creatine kinase (CK) levels were normal (85±18 U.I/l compared with 87 U.I./l in the healthy littermate).

**Fig. 2.**
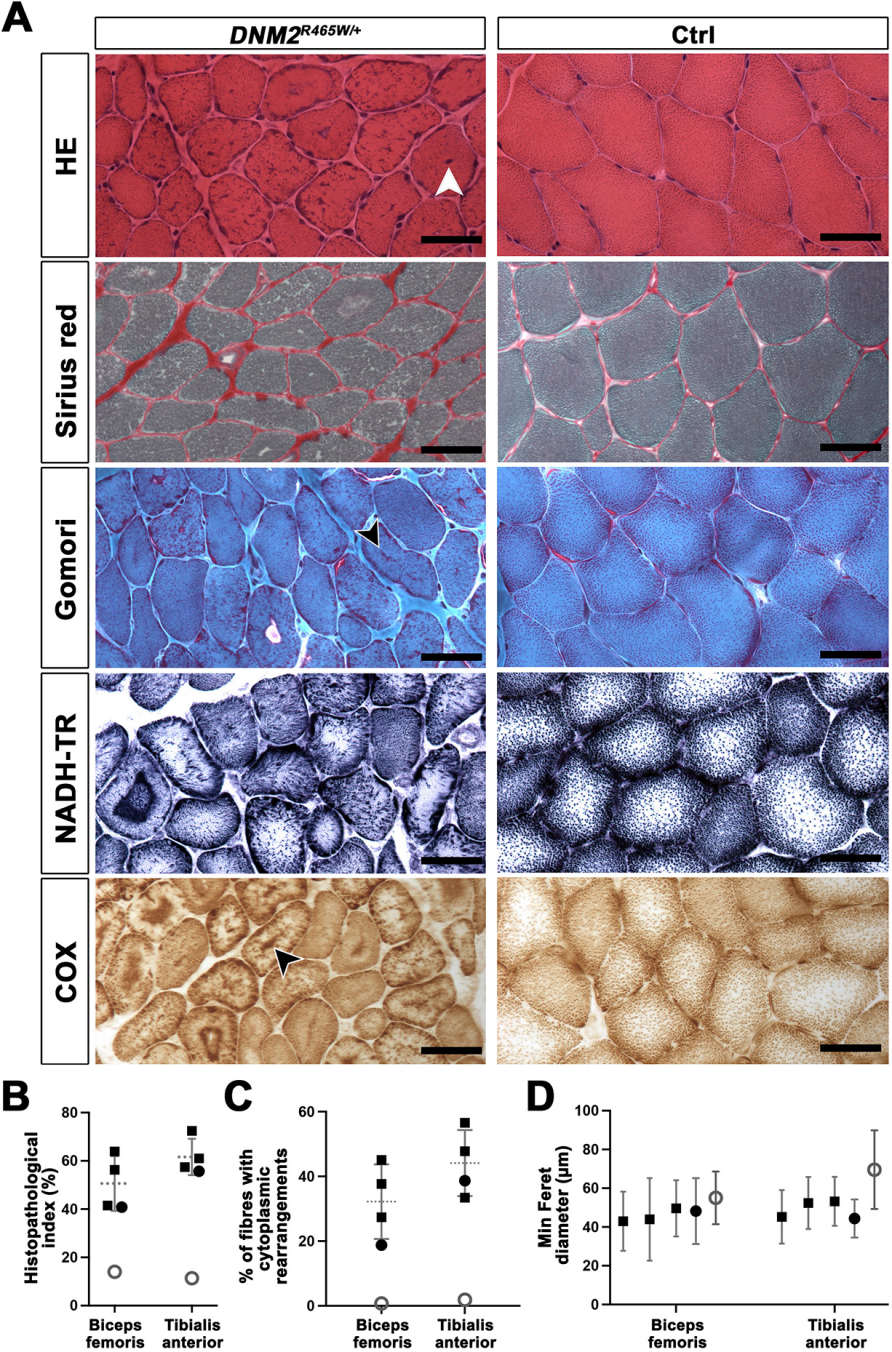
**Histological features of canine DNM2-CNM.** (A) Histological and histochemical analyses on transverse tibialis anterior sections at 12 months of age uncovered fibre atrophy and centralized nuclei (arrowhead) on H&E-stained sections, endomysial fibrosis on Sirius Red-stained sections, and prominent central or subsarcolemmal accumulations of mitochondria (arrowheads) on Gomori trichrome-, NADH-TR- and COX-stained sections in the affected dogs. Muscle samples from the healthy littermate served as control (Ctrl). Scale bars: 50 µm. (B) Histopathology index calculated on transverse sections of the biceps femoris and tibialis anterior muscles. (C) Percentage of fibres with cytoplasmic rearrangements calculated on transverse sections of the biceps femoris and tibialis anterior muscles. (D) Minimum Feret diameter of fibres calculated on transverse sections of the biceps femoris and tibialis anterior muscles. Black squares represent affected males, black circles the affected female, and white circles the healthy female littermate. The mean is represented by a dotted line in B,C and symbol position in D. Error bars represent s.d.

Histological and histochemical examinations of transverse sections of tibialis anterior and biceps femoris muscles disclosed fibre size variability, endomysial enlargement, and fibres with centralized nuclei in the *DNM2^R465W/+^* dogs (Fig. 2A). We also observed major cytoplasmic rearrangements, such as central and subsarcolemmal accumulations on Haematoxylin and Eosin (H&E), oxidative staining and COX assay, indicating a remodelled mitochondrial network in 19-57% of the myofibres (Fig. 2A-C). In addition, the fibre diameter was significantly reduced in the *DNM2^R465W/+^* dogs compared with the control littermate (Fig. 2D). Overall, and based on the cumulative anomalies on the muscle sections, the histopathology index was increased by a factor of three to five in *DNM2^R465W/+^* dogs (Fig. 2B).

Taken together, the clinical and histopathological features of the *DNM2^R465W/+^* dogs were highly consistent and conformed to the disease signs of the Border Collie male (Eminaga et al., 2012). Our data confirmed dominant disease transmission and the causality and full penetrance of the R465W mutation in the development of a mild and slowly progressive myopathy in dogs.

## DISCUSSION

The present study describes the first canine model for ADCNM, and the affected dogs carrying the *DNM2* c.1393C>T (R465W) mutation showed muscle weakness and ADCNM-typical morphological anomalies on muscle sections. Hence, we propose that *DNM2^R465W^*^/+^ dogs can be alternatively named DNM2-CNM dogs.

### R465W in humans, mice and dogs

In humans, *DNM2* mutations are the primary cause of ADCNM (Bitoun et al., 2005; Bohm et al., 2012), and investigations in cell and animal models suggest that the mutations involve a gain-of-function mechanism. Indeed, ADCNM-related DNM2 mutants have been shown to form oligomers with increased stability (Wang et al., 2010), and their exogenous expression in mice compromised skeletal muscle force and structure.

R465W is the most common dynamin 2 mutation. It has been reported in more than 30 families to date and is associated with inter- and intrafamilial variability (Bohm et al., 2012). Although the first signs of muscle weakness usually appear during childhood, patients with neonatal and adult disease onset and a varying degree of muscle weakness, facial weakness and eye movement defects have been described (Bohm et al., 2012). A *Dnm2^R465W/+^* mouse model also exists and exhibits a mildly progressive muscle weakness and atrophy from 3 weeks of age, and histological anomalies of the sarcoplasmic reticulum and mitochondrial distribution from 2 months of age (Durieux et al., 2010). Increased nuclear centralization is, however, not detectable in murine *Dnm2^R465W/+^* muscles, in contrast to the morphological muscle aberrations observed in DNM2-CNM dogs and patients. This may be related to the dissimilar muscle size and associated mechanical tension in the species, or might reflect physiological differences in muscle fibre development, maturation or maintenance, and highlights the importance of the DNM2-CNM dogs for further investigations on disease development and the underlying pathomechanisms.

CNM is a genetically heterogenous disease with X-linked, autosomal-dominant and autosomal-recessive forms essentially and respectively caused by mutations in *MTM1*, *DNM2* and *BIN1* (Bitoun et al., 2005; Bohm et al., 2014; Laporte et al., 1996; Nicot et al., 2007). Spontaneous canine *MTM1* and *BIN1* models have previously been reported, and all recapitulated the human disorders at the clinical and histopathological level with severe muscle atrophy, swallowing difficulties and a rapidly progressive tetraparesis. These major functional deficits required daily veterinary support and most often necessitated compassionate euthanasia between 3 and 6 months of age, preventing longitudinal studies (Beggs et al., 2010; Bohm et al., 2013; Olby et al., 2020; Shelton et al., 2015). Of note, none of the affected Labrador Retrievers, Rottweilers and Boykin Spaniels carried mutations found in patients. By contrast, the dogs described in the present study harbour the most common ADCNM mutation diagnosed in 25% of the patients, and the slowly progressive disease course enables a large panel of molecular investigations at different time points to decipher the aetiopathology and implicated pathways, and to identify relevant therapeutic targets for the prevention or reversal of the muscle phenotype.

### The importance of dogs for preclinical trials

Dogs represent valuable tools to complement the continuum of preclinical animal models dedicated to the establishment and evaluation of innovative therapeutic approaches in neuromuscular disorders (Barthelemy et al., 2019; Story et al., 2020). The downregulation of dynamin 2 through antisense oligonucleotides was shown to rescue the *DNM2*-related CNM phenotype in mice (Buono et al., 2018), and the application of this and other therapeutic strategies to a larger mammalian model with longer lifespan, such as the DNM2-CNM dogs, would provide important information on drug delivery options, pharmacokinetics, bioavailability, efficacy, durability and tolerability after sustained administration. In analogy, an AAV-mediated gene therapy for X-linked CNM (XLCNM) was first proven to be efficient on *Mtm1* knockout mice (Buj-Bello et al., 2008) and validated on a spontaneous canine XLCNM model (Childers et al., 2014) prior to its usage in clinical trials (NCT03199469). Moreover, dog models have served to establish exon skipping, genome editing and minigene expression strategies for Duchenne muscular dystrophy (Amoasii et al., 2018; Koo et al., 2011; Vulin et al., 2012), and have also been used for preclinical proof-of-concept studies of disorders affecting other tissues and organs.

In conclusion, the DNM2-CNM dog is a faithful model for the human disorder, allows longitudinal investigations to decipher the sequence of events leading to the muscle dysfunction, and represents an optimal complementary system to assess the safety and efficacy of therapeutic approaches before translation to humans.

## MATERIALS AND METHODS

### DNA analysis

Genomic DNA from a Border Collie male was prepared from peripheral blood by routine procedures, and Sanger sequenced for *HACD1* exon 2 and all coding exons and the adjacent splice elements of *MTM1*, *BIN1* and *DNM2*. The five offspring were Sanger sequenced for *DNM2* exon 11 for genotyping using forward (TGCTTGTCTCCCAGCTGCAG) and reverse (TGGTACCTTGACTGAGGTG) primers. The identified *DNM2* mutation was numbered according to GenBank XM_005632882.3 and XP_005632939.1.

### Ethics, animals and establishment of a colony

The establishment of the dog colony and animal experimentation were in accordance with European Community Standards and were performed following the acceptance of the project by the local EnvA-UPEC-ANSES ethical committee (approval number 13/02/18-1). The Ministère de l'enseignement supérieur, de la recherche et de l'innovation authorized the project (APAFIS #2018010910531134).

Sperm samples from the Border Collie male were collected in the UK with consent from the owner, and used to inseminate a Beagle female at EnvA, France. The resulting litter of two female and three male pups underwent regular clinical examinations and blood sampling. Muscle biopsies (biceps femoris and tibialis anterior muscles) were performed under a propofol-induced, isoflurane/morphine-maintained anaesthesia/analgesia.

### Muscle ultrasonography

The biceps femoris and sartorius cranialis muscles were longitudinally imaged using a linear 12.5-5 MHz transducer. Imaging depth was set at 4 cm, and muscle thickness was assessed with the internal measurement tool of an ultrasound Philips HD7 scanner (Philips, Amsterdam, The Netherlands). Echo intensity was determined in ImageJ as the mean grey level on the histogram of the muscle after drawing a region of interest. Three images were acquired per muscle and per dog, and the mean values were used for analysis.

### Accelerometry

Gait analysis was performed using 3D accelerometry as previously described (Barthelemy et al., 2009). Briefly, a device containing three orthogonally positioned accelerometers (Locometrix, Centaure Metrix, Evry, France) was inserted into a belt tightened around the thorax of the dogs. All dogs were evaluated at a trot. Accelerometric curves were acquired at 50 Hz along the cranio-caudal, dorso-ventral and medio-lateral gait axes, and data were analysed using Equimetrix^®^ software (Centaure Metrix) to calculate following parameters: stride length, stride frequency, speed, regularity, generated power for each axis, and total power. Generated power of each axis was expressed as percentage of the total power.

### Protein studies

For western blot, protein extracts from muscle samples were loaded on a 10% SDS PAGE, and membranes were incubated with rabbit anti-dynamin 2 (R2865; 1:700; Massana Munoz et al., 2019) and rabbit anti-calnexin (C-4731; Sigma-Aldrich; 1:1000), as well as with peroxidase-coupled goat anti-rabbit antibodies (111-035-144; Jackson ImmunoResearch; 1:10,000). The immunoblots were revealed with the SuperSignal West Pico kit (Thermo Fisher Scientific), and monitored on an Amersham Imager 600 (GE Healthcare Life Sciences). Quantification of band intensity was performed using the Measurement Log plugin of Adobe Photoshop 2022, version 23.1.0. The DNM2/calnexin ratio of the integrated grey level density was calculated for each sample and expressed as the percentage of the average ratio in control dogs. The individual ratio values were plotted using Prism 9 for MacOS, version 9.3.1.

### Muscle morphology

Biceps femoris and tibialis anterior muscle biopsies were taken at 12 months of age. Transverse sections (10 µm) were stained with H&E, Sirius Red, modified Gomori trichrome, NADH tetrazolium reductase (NADH-TR) and cytochrome c oxidase (COX), and assessed for fibre morphology, accumulation/infiltrations, and oxidative activity. The myofibre diameter was determined using an ImageJ-developed macro script (Reyes-Fernandez et al., 2019) and is defined as the minimum Feret on sections immunostained with a rabbit anti-caveolin antibody (ab173575; Abcam). Quantitative analyses were performed on H&E staining. Entire muscle sections were analysed using Visilog 7.0 software (Noesis). A grid was superimposed onto the image, and muscle morphology was assessed at each of the intercepts and manually annotated (1000 annotations per section) as previously described (Spencer et al., 2001). The histopathology index corresponds to the percentage of pathological features not corresponding to normal myofibres.

## References

[DMM049219C1] Abath Neto, O., Martins Cde, A., Carvalho, M., Chadi, G., Seitz, K. W., Oliveira, A. S., Reed, U. C., Laporte, J. and Zanoteli, E. (2015). DNM2 mutations in a cohort of sporadic patients with centronuclear myopathy. *Genet. Mol. Biol.* 38, 147-151. 10.1590/S1415-4757382220140238 26273216 PMC4530644

[DMM049219C2] Amoasii, L., Hildyard, J. C. W., Li, H., Sanchez-Ortiz, E., Mireault, A., Caballero, D., Harron, R., Stathopoulou, T. R., Massey, C., Shelton, J. M. et al. (2018). Gene editing restores dystrophin expression in a canine model of Duchenne muscular dystrophy. *Science* 362, 86-91. 10.1126/science.aau1549 30166439 PMC6205228

[DMM049219C3] Antonny, B., Burd, C., De Camilli, P., Chen, E., Daumke, O., Faelber, K., Ford, M., Frolov, V. A., Frost, A., Hinshaw, J. E. et al. (2016). Membrane fission by dynamin: what we know and what we need to know. *EMBO J.* 35, 2270-2284. 10.15252/embj.201694613 27670760 PMC5090216

[DMM049219C4] Barthelemy, I., Barrey, E., Thibaud, J. L., Uriarte, A., Voit, T., Blot, S. and Hogrel, J. Y. (2009). Gait analysis using accelerometry in dystrophin-deficient dogs. *Neuromuscul. Disord.* 19, 788-796. 10.1016/j.nmd.2009.07.014 19800232

[DMM049219C5] Barthelemy, I., Hitte, C. and Tiret, L. (2019). The dog model in the spotlight: legacy of a trustful cooperation. *J Neuromuscul Dis* 6, 421-451. 10.3233/JND-190394 31450509 PMC6918919

[DMM049219C6] Beggs, A. H., Bohm, J., Snead, E., Kozlowski, M., Maurer, M., Minor, K., Childers, M. K., Taylor, S. M., Hitte, C., Mickelson, J. R. et al. (2010). MTM1 mutation associated with X-linked myotubular myopathy in Labrador Retrievers. *Proc. Natl. Acad. Sci. USA* 107, 14697-14702. 10.1073/pnas.1003677107 20682747 PMC2930454

[DMM049219C7] Bitoun, M., Maugenre, S., Jeannet, P. Y., Lacene, E., Ferrer, X., Laforet, P., Martin, J. J., Laporte, J., Lochmuller, H., Beggs, A. H. et al. (2005). Mutations in dynamin 2 cause dominant centronuclear myopathy. *Nat. Genet.* 37, 1207-1209. 10.1038/ng1657 16227997

[DMM049219C8] Bohm, J., Biancalana, V., Dechene, E. T., Bitoun, M., Pierson, C. R., Schaefer, E., Karasoy, H., Dempsey, M. A., Klein, F., Dondaine, N. et al. (2012). Mutation spectrum in the large GTPase dynamin 2, and genotype-phenotype correlation in autosomal dominant centronuclear myopathy. *Hum. Mutat.* 33, 949-959. 22396310 10.1002/humu.22067 PMC3374402

[DMM049219C9] Bohm, J., Biancalana, V., Malfatti, E., Dondaine, N., Koch, C., Vasli, N., Kress, W., Strittmatter, M., Taratuto, A. L., Gonorazky, H. et al. (2014). Adult-onset autosomal dominant centronuclear myopathy due to BIN1 mutations. *Brain* 137, 3160-3170. 10.1093/brain/awu272 25260562

[DMM049219C10] Bohm, J., Vasli, N., Maurer, M., Cowling, B., Shelton, G. D., Kress, W., Toussaint, A., Prokic, I., Schara, U., Anderson, T. J. et al. (2013). Altered splicing of the BIN1 muscle-specific exon in humans and dogs with highly progressive centronuclear myopathy. *PLoS Genet.* 9, e1003430. 10.1371/journal.pgen.1003430 23754947 PMC3675003

[DMM049219C11] Buj-Bello, A., Fougerousse, F., Schwab, Y., Messaddeq, N., Spehner, D., Pierson, C. R., Durand, M., Kretz, C., Danos, O., Douar, A. M. et al. (2008). AAV-mediated intramuscular delivery of myotubularin corrects the myotubular myopathy phenotype in targeted murine muscle and suggests a function in plasma membrane homeostasis. *Hum. Mol. Genet.* 17, 2132-2143. 10.1093/hmg/ddn112 18434328 PMC2441725

[DMM049219C12] Buono, S., Ross, J. A., Tasfaout, H., Levy, Y., Kretz, C., Tayefeh, L., Matson, J., Guo, S., Kessler, P., Monia, B. P. et al. (2018). Reducing dynamin 2 (DNM2) rescues DNM2-related dominant centronuclear myopathy. *Proc. Natl. Acad. Sci. USA* 115, 11066-11071. 10.1073/pnas.1808170115 30291191 PMC6205463

[DMM049219C13] Chappie, J. S., Acharya, S., Liu, Y. W., Leonard, M., Pucadyil, T. J. and Schmid, S. L. (2009). An intramolecular signaling element that modulates dynamin function in vitro and in vivo. *Mol. Biol. Cell* 20, 3561-3571. 10.1091/mbc.e09-04-0318 19515832 PMC2719574

[DMM049219C14] Childers, M. K., Joubert, R., Poulard, K., Moal, C., Grange, R. W., Doering, J. A., Lawlor, M. W., Rider, B. E., Jamet, T., Daniele, N. et al. (2014). Gene therapy prolongs survival and restores function in murine and canine models of myotubular myopathy. *Sci. Transl. Med.* 6, 220ra10. 10.1126/scitranslmed.3007523 PMC4105197 24452262

[DMM049219C15] Durieux, A. C., Vignaud, A., Prudhon, B., Viou, M. T., Beuvin, M., Vassilopoulos, S., Fraysse, B., Ferry, A., Laine, J., Romero, N. B. et al. (2010). A centronuclear myopathy-dynamin 2 mutation impairs skeletal muscle structure and function in mice. *Hum. Mol. Genet.* 19, 4820-4836. 10.1093/hmg/ddq413 20858595

[DMM049219C16] Eminaga, S., Cherubini, G. B. and Shelton, G. D. (2012). Centronuclear myopathy in a Border collie dog. *J. Small Anim. Pract.* 53, 608-612. 10.1111/j.1748-5827.2012.01265.x 23013377

[DMM049219C17] Faelber, K., Posor, Y., Gao, S., Held, M., Roske, Y., Schulze, D., Haucke, V., Noe, F. and Daumke, O. (2011). Crystal structure of nucleotide-free dynamin. *Nature* 477, 556-560. 10.1038/nature10369 21927000

[DMM049219C18] Ferguson, S. M. and De Camilli, P. (2012). Dynamin, a membrane-remodelling GTPase. *Nat. Rev. Mol. Cell Biol.* 13, 75-88. 10.1038/nrm3266 22233676 PMC3519936

[DMM049219C19] Gu, C., Yaddanapudi, S., Weins, A., Osborn, T., Reiser, J., Pollak, M., Hartwig, J. and Sever, S. (2010). Direct dynamin-actin interactions regulate the actin cytoskeleton. *EMBO J.* 29, 3593-3606. 10.1038/emboj.2010.249 20935625 PMC2982766

[DMM049219C20] Koo, T., Okada, T., Athanasopoulos, T., Foster, H., Takeda, S. and Dickson, G. (2011). Long-term functional adeno-associated virus-microdystrophin expression in the dystrophic CXMDj dog. *J. Gene Med.* 13, 497-506. 10.1002/jgm.1602 22144143

[DMM049219C21] Laporte, J., Hu, L. J., Kretz, C., Mandel, J. L., Kioschis, P., Coy, J. F., Klauck, S. M., Poustka, A. and Dahl, N. (1996). A gene mutated in X-linked myotubular myopathy defines a new putative tyrosine phosphatase family conserved in yeast. *Nat. Genet.* 13, 175-182. 10.1038/ng0696-175 8640223

[DMM049219C22] Massana Munoz, X., Buono, S., Koebel, P., Laporte, J. and Cowling, B. S. (2019). Different in vivo impacts of dynamin 2 mutations implicated in Charcot-Marie-Tooth neuropathy or centronuclear myopathy. *Hum. Mol. Genet.* 28, 4067-4077. 10.1093/hmg/ddz249 31628461

[DMM049219C23] McMahon, H. T. and Gallop, J. L. (2005). Membrane curvature and mechanisms of dynamic cell membrane remodelling. *Nature* 438, 590-596. 10.1038/nature04396 16319878

[DMM049219C24] Nicot, A. S., Toussaint, A., Tosch, V., Kretz, C., Wallgren-Pettersson, C., Iwarsson, E., Kingston, H., Garnier, J. M., Biancalana, V., Oldfors, A. et al. (2007). Mutations in amphiphysin 2 (BIN1) disrupt interaction with dynamin 2 and cause autosomal recessive centronuclear myopathy. *Nat. Genet.* 39, 1134-1139. 10.1038/ng2086 17676042

[DMM049219C25] Olby, N. J., Friedenberg, S., Meurs, K., DeProspero, D., Guevar, J., Lau, J., Yost, O., Guo, L. T. and Shelton, G. D. (2020). A mutation in MTM1 causes X-Linked myotubular myopathy in Boykin spaniels. *Neuromuscul. Disord.* 30, 353-359. 10.1016/j.nmd.2020.02.021 32417001 PMC7532942

[DMM049219C26] Pele, M., Tiret, L., Kessler, J. L., Blot, S. and Panthier, J. J. (2005). SINE exonic insertion in the PTPLA gene leads to multiple splicing defects and segregates with the autosomal recessive centronuclear myopathy in dogs. *Hum. Mol. Genet.* 14, 1417-1427. 10.1093/hmg/ddi151 15829503

[DMM049219C27] Reyes-Fernandez, P. C., Periou, B., Decrouy, X., Relaix, F. and Authier, F. J. (2019). Automated image-analysis method for the quantification of fiber morphometry and fiber type population in human skeletal muscle. *Skelet. Muscle* 9, 15. 10.1186/s13395-019-0200-7 31133066 PMC6537183

[DMM049219C28] Sambuughin, N., Goldfarb, L. G., Sivtseva, T. M., Davydova, T. K., Vladimirtsev, V. A., Osakovskiy, V. L., Danilova, A. P., Nikitina, R. S., Ylakhova, A. N., Diachkovskaya, M. P. et al. (2015). Adult-onset autosomal dominant spastic paraplegia linked to a GTPase-effector domain mutation of dynamin 2. *BMC Neurol.* 15, 223. 10.1186/s12883-015-0481-3 26517984 PMC4628244

[DMM049219C29] Shelton, G. D., Rider, B. E., Child, G., Tzannes, S., Guo, L. T., Moghadaszadeh, B., Troiano, E. C., Haase, B., Wade, C. M. and Beggs, A. H. (2015). X-linked myotubular myopathy in Rottweiler dogs is caused by a missense mutation in exon 11 of the MTM1 gene. *Skelet. Muscle* 5, 1. 10.1186/s13395-014-0025-3 25664165 PMC4320619

[DMM049219C30] Spencer, M. J., Montecino-Rodriguez, E., Dorshkind, K. and Tidball, J. G. (2001). Helper (CD4(+)) and cytotoxic (CD8(+)) T cells promote the pathology of dystrophin-deficient muscle. *Clin Immunol* 98, 235-243. 10.1006/clim.2000.4966 11161980

[DMM049219C31] Story, B. D., Miller, M. E., Bradbury, A. M., Million, E. D., Duan, D., Taghian, T., Faissler, D., Fernau, D., Beecy, S. J. and Gray-Edwards, H. L. (2020). Canine models of inherited musculoskeletal and neurodegenerative diseases. *Front. Vet. Sci.* 7, 80. 10.3389/fvets.2020.00080 32219101 PMC7078110

[DMM049219C32] Vulin, A., Barthelemy, I., Goyenvalle, A., Thibaud, J. L., Beley, C., Griffith, G., Benchaouir, R., le Hir, M., Unterfinger, Y., Lorain, S. et al. (2012). Muscle function recovery in golden retriever muscular dystrophy after AAV1-U7 exon skipping. *Mol. Ther.* 20, 2120-2133. 10.1038/mt.2012.181 22968479 PMC3498802

[DMM049219C33] Wang, L., Barylko, B., Byers, C., Ross, J. A., Jameson, D. M. and Albanesi, J. P. (2010). Dynamin 2 mutants linked to centronuclear myopathies form abnormally stable polymers. *J. Biol. Chem.* 285, 22753-22757. 10.1074/jbc.C110.130013 20529869 PMC2906265

[DMM049219C34] Warnock, D. E., Baba, T. and Schmid, S. L. (1997). Ubiquitously expressed dynamin-II has a higher intrinsic GTPase activity and a greater propensity for self-assembly than neuronal dynamin-I. *Mol. Biol. Cell* 8, 2553-2562. 10.1091/mbc.8.12.2553 9398675 PMC25727

[DMM049219C35] Werlauff, U., Petri, H., Witting, N. and Vissing, J. (2015). Frequency and phenotype of myotubular myopathy amongst danish patients with congenital myopathy older than 5 years. *J. Neuromuscul. Dis.* 2, 167-174. 10.3233/JND-140040 27858727 PMC5271486

[DMM049219C36] Zuchner, S., Noureddine, M., Kennerson, M., Verhoeven, K., Claeys, K., De Jonghe, P., Merory, J., Oliveira, S. A., Speer, M. C., Stenger, J. E. et al. (2005). Mutations in the pleckstrin homology domain of dynamin 2 cause dominant intermediate Charcot-Marie-Tooth disease. *Nat. Genet.* 37, 289-294. 10.1038/ng1514 15731758

